# Thoracic Kyphosis is Now Uncommon Amongst Children and Adolescents with Cystic Fibrosis

**DOI:** 10.3389/fped.2014.00011

**Published:** 2014-02-17

**Authors:** Nicki Barker, Ashok Raghavan, Pauline Buttling, Kostas Douros, Mark Lloyd Everard

**Affiliations:** ^1^Physiotherapy Department, Sheffield Children’s Hospital, Sheffield, UK; ^2^Department of Radiology, Sheffield Children’s Hospital, Sheffield, UK; ^3^Faculty of Health and Wellbeing, Sheffield Hallam University, Sheffield, UK; ^4^Department of Respiratory Medicine, Sheffield Children’s Hospital, Sheffield, UK

**Keywords:** thoracic kyphosis, cystic fibrosis, children, Cobb method, lung function

## Abstract

Historically, thoracic kyphosis has been reported to be common amongst patients with cystic fibrosis (CF). The mechanisms leading to the development of this abnormality of the chest wall are not fully understood. In order to explore the prevalence of the condition amongst children with CF in the early twenty-first century and to explore factors that might be contributing to its development, a retrospective cross sectional study was undertaken in a regional CF unit. Data were obtained from 74 children with CF aged 8–16 years attending for their annual review. Thoracic kyphosis was measured from lateral chest X-ray using an alternative Cobb method. Lung function, disease severity, and nutritional status were also recorded. Correlations between measures were explored using a multiple linear regression model. The range of Cobb angles measured was 5.4–44.3° with thoracic kyphosis identified in only two subjects. There was no correlation between age and thoracic kyphosis, however, there was a significant correlation between lung function and thoracic kyphosis (*p* = 0.004). Regression coefficient (*b*) was −0.26 (95% CI: −0.44, −0.08). The prevalence of thoracic kyphosis is significantly less amongst children with CF than previously reported. This appears likely to be associated with the overall improvements in pulmonary status. Studies of older populations may bring further understanding of increasing thoracic kyphosis in people with CF.

## Introduction

The life expectancy of people with cystic fibrosis (CF) continues to improve, having increased significantly over the last 50 years (1). This increase in life expectancy is associated with an increased incidence of CF related conditions such as liver disease, diabetes, and musculoskeletal disorders, including postural problems.

The most common postural problem for people with CF is thoracic kyphosis. Though the development of thoracic kyphosis in patients with CF has received relatively little attention, previous work has suggested that increased kyphosis is relatively common in this population (2–4). Parasa and Maffulli (5), in their review of musculoskeletal problems in CF, reported that up to 62% of patients had excessive thoracic kyphosis. The development of an abnormal thoracic kyphosis appears to be associated with significant morbidity, back pain, poor body image, and low mood, all impacting on quality of life (6).

The majority of studies investigating thoracic kyphosis in CF have been undertaken in adults and hence the role of age is unclear. One study of children and young adults undertaken in the 1970s suggested that increased thoracic kyphosis was observed in those as young as 5 years of age and that the prevalence of thoracic kyphosis increased with increasing age (2). However, management has improved significantly over the past decades and it is unclear whether these finding are relevant to the current cohort of children and young people with CF.

Understanding the development of thoracic kyphosis in children and adolescents with CF may help to develop both preventative and therapeutic strategies to mitigate the associated morbidity and, potentially, the possible negative effects on disease progression. In order to obtain information relevant to children and young people currently attending CF clinics, an open cross sectional study was undertaken in patients aged 8–16 years. The aim of this study was to investigate the prevalence of abnormal thoracic kyphosis and the factors that may contribute to its development.

## Materials and Methods

The study was undertaken using routine data collected from patients aged 8–16 years of age attending for annual review at the Sheffield Children’s Hospital CF center. Data collected as part of the annual review were obtained for 74 subjects with confirmed CF and included lateral chest X-rays and spirometry. Children were excluded if they had a known congenital spinal deformity or were not capable of performing lung function tests.

Thoracic kyphosis was measured using an alternative Cobb method (7) performed on lateral chest X-rays via the computer based picture archiving and communication system (PACS), Agfa version 6.2. The alternative Cobb method was performed by identifying the vertebrae T4 and T9. A line was then drawn through the center of each of the two identified vertebral bodies, i.e., parallel to the side walls of the vertebra, with the angle of intersection of the lines giving the Cobb angle. All Cobb method calculations were performed by a single researcher and executed in the order of the original alphabetical sample list.

Assessment of intra- and inter-rater reliability was undertaken. For this, 20% of the original sample was randomly selected a minimum of 2 weeks after the original calculations were made. A non-involved party picked 16 subjects, identified solely by number, from the sample list. The researcher then repeated calculations for 8 of these subjects whilst a consultant radiologist carried out calculations for the remaining 8.

Lung function was obtained using a Jaeger MasterScope Spirometer (Cardinal Health) following the ATS/ERS guidelines (8) with all children standing and using a nose clip. Shwachman scores (as a measure of disease severity) were calculated by the lead clinician as part of the annual review and independent of this study (9). Nutritional status was assessed with the use of body mass index (BMI) *z*-score.

Statistical analysis was performed using the Statistical Package for Social Sciences (SPSS) version 15 with *p* < 0.05. For multivariate analysis, a multiple linear regression model was used. Cobb angle was the dependent variable and age, gender, FEV_1_, FVC, Shwachman score, and BMI *z*-score were the independent variables. Inter- and intra-rater reliability were assessed using intraclass correlation coefficients.

Ethical approval was not required due to the retrospective nature of the study design and the inability to identify individuals.

## Results

Of 83 eligible subjects, 56 full and 18 partial sets of data were obtained on 74 subjects. Nine subjects did not attend for an annual review within the 12-month period under consideration. Of those with partial data, Shwachman score was unavailable in 16 subjects and in 4 subjects it was not possible to calculate the Cobb angle due to inability to identify the superior aspect of T4.

Table 1 is a summary of the data collected subdivided by gender. Amongst the subjects, 2 female subjects had significantly lower values for lung function parameters compared to all other subjects with FEV_1_ below 40% predicted. Statistical analysis was performed both with and without these outlying results; small differences were seen but there was limited effect on the overall relationships.

**Table 1:** **Summary of data subdivided by gender**.

Gender		*n*	Median	25h^th^ Percentile	75h^th^ Percentile	IQR
F (*n* = 44)	Age (years)	41	11.8	9.8	13.4	3.5
	Cobb angle (°)	42	20.1	12.5	26.5	13.7
	FEV_1_ (%)	40	93.0	79.0	99.7	19.5
	FVC (%)	40	100.3	87.3	113.9	20.6
	Shwachman score	36	77.5	75	83	8
	BMI *z*-score	41	−0.28	−0.85	0.54	1.42
M (*n* = 30)	Age (years)	23	12.1	10.3	14.9	4.6
	Cobb angle (°)	29	17.8	13.5	24.1	10.6
	FEV_1_ (%)	24	93.6	82.2	98.1	15.9
	FVC (%)	24	99.6	93.4	108.4	15.0
	Shwachman score	22	81	76	83	7
	BMI *z*-score	23	−0.08	−1.07	0.24	1.32

Using a conservative upper limit for normal thoracic kyphosis of 35°, 2 subjects were identified as having an abnormal thoracic kyphosis. This number decreased to 1 subject if a higher limit of 40° was chosen. The prevalence of abnormal thoracic kyphosis was therefore either 2.8 or 1.4% respectively depending on the chosen upper limit of normal (as shown in Table 2). One of the subjects with abnormal thoracic kyphosis was also one of the 2 females with particularly low lung function values.

**Table 2:** **Comparison of prevalence of abnormal thoracic kyphosis**.

Upper limits for normal thoracic kyphosis	Prevalence of abnormal thoracic kyphosis (%)			
	Current study	Erkkila et al. (2)	Denton et al. (3)	Logvinoff et al. (4)
35°	2.8 (aged 8–16)	14 (aged 5–9)		
		32 (aged 10–14)	
40°	1.4 (aged 8–16)		5.5 (adolescents)	
Mean + 2 SD	2.8 (aged 8–16)			22 (aged 6–36)

**Figure 1:**
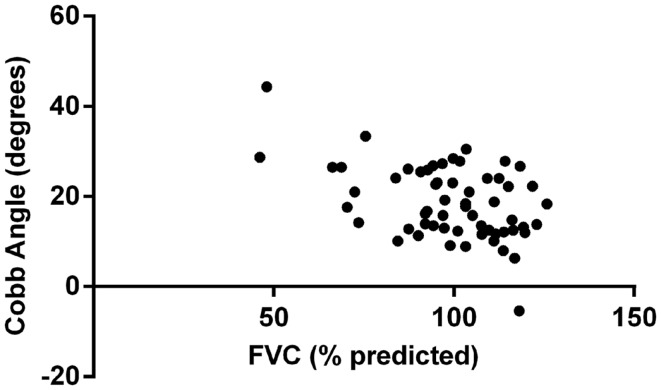
**Correlation between Cobb angle and FVC**.

**Figure 2:**
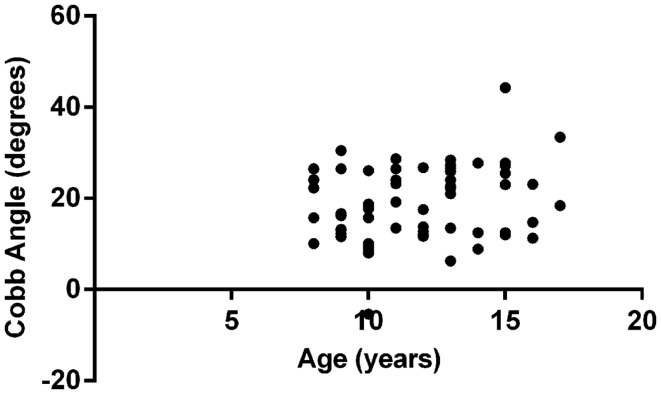
**Lack of correlation between Cobb angle and age**.

Multivariate analysis showed that FVC was significantly correlated with Cobb angle (*p* = 0.004), as shown in Figure 1. Regression coefficient (*b*) was −0.26 (95% CI: −0.44, −0.08) implying that for every 1% unit decrease in FVC the mean increase in Cobb angle is 0.26°. None of the other variables included in the model (age, gender, FEV_1_, Shwachman score, and BMI *z*-score) reached statistical significance. Figure 2 illustrates the lack of correlation between age and Cobb angle.

The outcomes of the intraclass correlation coefficients for inter- and intra-rater reliability are 0.976 and 0.979, respectively. Similar results for Cobb angle were achieved when an uninvolved, experienced consultant radiologist and the researcher repeated measurements.

## Discussion

The results indicate that, with current management, thoracic kyphosis is now uncommon amongst children and adolescents with CF. Even with the most stringent criteria, using a Cobb angle of >35°, less than 3% of children exhibited this postural abnormality. This compares favorably with data from studies undertaken in the 1980s and 1990s (see Table 2), which found significantly higher prevalence of this condition amongst CF patients within the same age group. The results also indicate that, in this age group, there is no significant relationship between increasing age and the development of thoracic kyphosis.

Whilst the mechanism for the development of abnormal thoracic kyphosis remains unconfirmed, studies have demonstrated that all muscles of the trunk have dual roles in respiration and postural support (10–12). The implication of this work is that if increasing demands are placed on one function, the other cannot be carried out effectively. Massery (1) proposed that the increased coughing associated with CF lung disease (and the postures assumed during this activity) places abnormal outward pressure on the spine, which leads to thoracic kyphosis (and secondary mal-alignment of the shoulder girdle).

It has also been suggested, conversely, that thoracic kyphosis may contribute to poor lung function and impaired clearance of secretions through altered chest mechanics resulting from structural changes of the spine and rib cage (5, 6). A vicious cycle may develop with deteriorating posture, lung function, and overall health and wellbeing. However, the evidence base for this is not robust and the suggestion that the chest deformities may in themselves accelerate the rate of decline in lung function is the subject of on-going debate (2–4, 6, 13).

This study does however provide some support for the suggestion that the development of thoracic kyphosis in patients with CF is related, at least in part, to deteriorating lung function though this cross sectional study is unable to determine cause and effect. This cohort had few individuals with significantly impaired lung function and mean spirometric indices were within normal limits. Other indices of severity also supported the suggestion that most subjects had mild disease with the mean Shwachman score >77 and normal nutritional status.

The significant reduction in reported thoracic kyphosis when compared to historical reports, together with the improved clinical status of patients, provides further support for the suggestion that disease severity contributes significantly to the development of thoracic kyphosis. This would indicate that, in the majority of cases, the current level of postural intervention, which involves monitoring for postural change, education regarding the importance of good posture, and advice on how to incorporate good posture into normal daily activities is appropriate.

Despite the fact that thoracic kyphosis was uncommon, there were 2 outliers with evidence of the condition. The subject with the highest Cobb angle had the most severe disease but also received less frequent and less supervised CF care in comparison to all other cases (due to geographical and socioeconomic factors). Hence while vigilance and appropriate advice remain important, our experience would suggest that optimization of care and prevention of severe disease is a key component in preventing the development of this abnormality.

This study suggests that, for most patients with CF, significant thoracic kyphosis is unlikely to occur until adulthood (if it develops at all) and hence future studies should be undertaken in this population. Despite being considered the “gold standard” for the measurement of thoracic kyphosis (14), the Cobb method has some weaknesses that may be more evident in an adult population (6, 14–16). The primary problem is the effect of altered end plate angle on the measured kyphotic angle. This is thought to be minimal in children but may become more of an issue for subjects of increasing age. A further consideration is that a non-fixed range for thoracic kyphosis may be more appropriate in analyses involving adults. A single upper limit of “normality” does not take into account the normal variation in thoracic kyphosis with age and this may generate some false abnormals in older age groups (17).

For this study, the ethical issue of exposing children unnecessarily to radiation from X-rays prevented the inclusion of a control group. However, it may be possible to collect data on chest wall structure and function in normal children and adults in the future using new developments in non-radiological measurement techniques such as topographical scanning (the use of light to measure physical form).

In conclusion, this study demonstrates low levels of thoracic structural abnormalities amongst children and adolescents and hence, while remaining vigilant and providing advice regarding postural issues, it appears unnecessary to focus specifically on thoracic kyphosis in individuals with mild disease.

## Author Contributions

Design of study was done by Nicki Barker, Pauline Buttling, Ashok Raghavan; collection and analysis of data was carried out by Nicki Barker, Ashok Raghavan; statistical analysis was done by Nicki Barker, Kostas Douros; drafting and revising the paper was done by Nicki Barker, Ashok Raghavan, Pauline Buttling, Kostas Douros, and Mark L. Everard.

## Conflict of Interest Statement

The authors declare that the research was conducted in the absence of any commercial or financial relationships that could be construed as a potential conflict of interest.
